# Supplementary material for: The impact of task-sharing scalable mental health interventions on non-specialist providers: a scoping review
Source: Glob Ment Health (Camb). 2025 Jan 13;11:e134. doi: 10.1017/gmh.2024.129 (PMC11729486; doi:10.1017/gmh.2024.129)

**Search Strategy**

The search strategy provided is the outline of all the steps taken for searches conducted by the first two authors, across multiple databases, the results obtained, the criteria for stopping the search, reasons for exclusion, and the final number of results found for review via the search query.

The search was continued till the point when additional searches resulted in no new or relevant studies. Some search queries were specifically filtered to only include studies conducted in the past 20 years to manage the scope and high volume of the search results. Furthermore, it allowed the authors to analyze the most relevant and up-to-date studies. The aim of this criteria was to ensure that the findings were directly relevant to the current task-sharing mental health interventions.

Reference sections of included articles were also scanned manually by authors to identify any additional studies that were relevant. Out of the final search query results, papers were further manually shortlisted according to how relevant they were based on the inclusion criteria. These papers were then thoroughly reviewed by the authors to create the final list of included studies based on how closely they complied to the inclusion criteria. If any of the exclusion criteria were found during the review of the full length paper, it was removed from the shortlist. No external software was used for selecting relevant studies.

The final number of studies that were used for the scoping review underwent several rounds of pre-defined checks such as focus on task-sharing mental health interventions and consisting relevant information about their impact on non-specialists and their quality of data. Their characteristics were recorded in detail to ensure they met the standards for inclusion and relevance. For the purposes of replication for future studies, a screenshot of the table is attached at the end of this document for additional supplementary information.

Timeline

The database searches were conducted from May 30, 2023 to July 20, 2023.

Databases Searched

1. PubMed
2. NLM
3. ScienceDirect
4. Biomedcentral

Filters Applied
Publication Filter: Studies conducted within the last 20 years.

Search Strategy for PubMed

Search Terms:

1. (task-sharing OR mental health) AND (non-specialists OR facilitators OR non-specialists OR paraprofessionals). ti,ab,kw (title, abstract, keyword).
   1. Search Results: 2,119
   2. Results After Publication Filter: 1094
   3. Search Stopping Criteria: Search stopped at Page 10 (100 results)
   4. Reasons for Exclusion: No mention of task-sharing mental health interventions and/or no mention of non-specialists in title or abstract in subsequent pages
   5. Final Number of Studies in Search Query: 100
2. (task-sharing OR mental health care) AND (paraprofessionals OR community health workers). ti,ab,kw.
   1. Search Results: 34,798
   2. Search Stopping Criteria: Search stopped at Page 5
   3. Reasons for Exclusion: No mention of task-sharing mental health interventions and/or no mention of non-specialists in title or abstract in subsequent pages
   4. Final Number of Studies in Search Query: 50
3. (task-sharing) AND (social health workers). ti,ab,kw.
   1. Search Results: 17
   2. Search Stopping Criteria: All results reviewed
   3. Final Number of Studies in Search Query: 17
4. (task-sharing) AND (facilitators OR social workers OR non-specialists OR non-specialist providers OR helpers OR paraprofessionals OR lay providers OR lay professionals OR community workers OR community health workers peer volunteers). ti,ab,kw.
   1. Search Results: 215,628
   2. Results After Publication Filter: 110,397
   3. Search Stopping Criteria: Search stopped at Page 4
   4. Reasons for Exclusion: No mention of task-sharing mental health interventions and/or no mention of non-specialists in title or abstract in subsequent pages
   5. Final Number of Studies in Search Query: 40
5. (“impact of task-sharing intervention on facilitators”).ti,ab,kw.
   1. Search Results: 9
   2. Search Stopping Criteria: All results viewed
   3. Final Number of Studies in Search Query: 9
6. (“mental healthcare by layworkers”).ti,ab,kw.
   1. Search Results: 304
   2. Search Stopping Criteria: All results viewed
   3. Final Number of Studies in Search Query: 304
7. (“layworkers experience in mental health care”).ti,ab,kw.
   1. Search Results: 57
   2. Search Stopping Criteria: All results viewed
   3. Final Number of Studies in Search Query: 57
8. (“benefits of task-sharing on providers”).ti,ab,kw.
   1. Search Results: 42
   2. Search Stopping Criteria: All results viewed
   3. Final Number of Studies in Search Query: 42

Search Strategy for NLM

Search Terms:

1. (“impact on paraprofessionals”).ti,ab,kw.
   1. Search Results: 206
   2. Search Stopping Criteria: All results viewed
   3. Final Number of Studies in Search Query: 206
2. (“mental health care by paraprofessionals”).ti,ab,kw.
   1. Search Results: 187
   2. Search Stopping Criteria: All results viewed
   3. Final Number of Studies in Search Query: 187
3. (“mental health care by layworkers”).ti,ab,kw.
   1. Search Results: 4
   2. Search Stopping Criteria: All results viewed
   3. Final Number of Studies in Search Query: 4

Search Strategy for ScienceDirect

Search Terms:

1. (“impact of mental health care”) AND (non specialists).ti,ab,kw.
   1. Search Results: 30,898
   2. Search Stopping Criteria: Search stopped at Page 4
   3. Reasons for Exclusion: No mention of task-sharing interventions after Page 3
   4. Final Number of Studies in Search Query: 100
2. Filter: subject area-“psychology”/(“impact of task sharing on paraprofessionals”).ti,ab,kw.
   1. Search Results: 398
   2. Search Stopping Criteria: Search stopped at Page 4
   3. Reasons for Exclusion: No mention of task-sharing interventions after Page 3
   4. Final Number of Studies in Search Query: 100
3. (paraprofessionals).ti,ab,kw.
   1. Search Results: 1,574
   2. Search Stopping Criteria: Search stopped at Page 4
   3. Reasons for Exclusion: No mention of task-sharing interventions after Page 3
   4. Final Number of Studies in Search Query: 100
4. (“task-shifting interventions”).ti,ab,kw.
   1. Search Results: 24,056
   2. Search Stopping Criteria: Search stopped at Page 4
   3. Reasons for Exclusion: No mention of task-sharing interventions after Page 3
   4. Final Number of Studies in Search Query: 100
5. (community health workers OR CHW).ti,ab,kw.
   1. Search Results: 243
   2. Search Stopping Criteria: All results viewed
   3. Final Number of Studies in Search Query: 243
6. (lay mental health workers).ti,ab,kw.
   1. Search Results: 2,640
   2. Search Stopping Criteria: Search stopped at Page 4
   3. Reasons for Exclusion: No mention of task-sharing interventions after Page 3
   4. Final Number of Studies in Search Query: 100
7. Filter: subject area-psychology/keyword-“task-shifting” (lay health workers).ti,ab,kw
   1. Search Results: 120
   2. Search Stopping Criteria: All results viewed
   3. Final Number of Studies in Search Query: 120

Search Strategy for Biomedcentral

Search Terms:

1. (“impact of mental health task-shifting on community health workers”).ti,ab,kw.
   1. Search Results: 438
   2. Search Stopping Criteria: All results viewed
   3. Final Number of Studies in Search Query: 438

Total Search Query Results: 313,738

Final Number of Included Studies After Search Exclusions: 2,317

Additional Supplementary Information

*Figure 1: Example of Search Strategy Table for Database Searches*
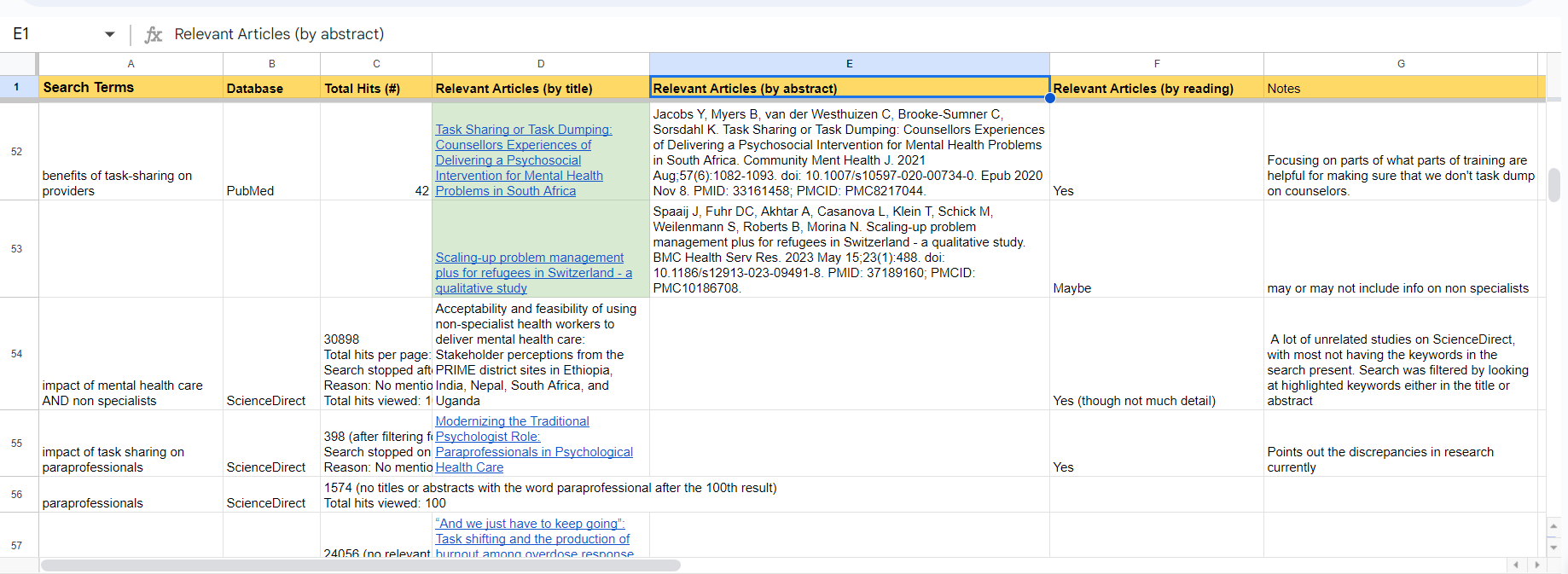

Supplement: Sangraula et al. supplementary material 2 — Sangraula et al. supplementary material [file S2054425124001298sup002.docx]
